# A novel risk factor of contrast associated acute kidney injury in patients after enhanced computed tomography: a retrospective study

**DOI:** 10.7717/peerj.14224

**Published:** 2022-10-20

**Authors:** You-Qi Li, Yongjun Shi, Wen-feng Deng, Shaobin Xiao, Wenwen Hu, Chengwen Huang, Xun Tang, Jun Zhang

**Affiliations:** 1 Nephrology, Zhujiang Hospital, Southern Medical University, GuangZhou, GuangDong, China; 2 Nephrology, Huizhou Central People‘s Hospital, Huizhou, Guangdong, China; 3 Huizhou Center for Disease Control and Prevention, Huizhou, Guangdong, China

**Keywords:** Red blood cell distribution width, Computed tomography, Contrast associated acute kidney injury

## Abstract

**Background:**

Contrast associated acute kidney injury (CA-AKI) is a major cause of acute renal failure and the incidence of CA-AKI is still high in recent years. Risk stratification is traditionally based on glomerular filtration rate(GFR). Hence, the aim of this study was to explore the novel risk factors for CA-AKI after enhanced computed tomography (CT).

**Methods:**

A retrospective cohort study was conducted in 632 in-hospital patients undergoing enhanced CT. The patients were divided into CA-AKI and no-CA-AKI groups. For comparative analyses, we applied one-to-four cohorts of those two groups using propensity score-matching methods addressing the imbalances of age, gender, weight, and smoking. The baseline clinical and biochemical data were compared. Logistic regression analysis was employed to investigate the CA-AKI risk factors. The receiver operating characteristic (ROC) curve was adopted to test the value of RDW in predicting CA-AKI after enhanced CT.

**Results:**

25 (3.96%) patients suffered from CA-AKI. Those subjects who developed CA-AKI had advanced age, severer renal functional injury, lower albumin, higher baseline RDW, neutrophil to lymphocyte ratio (NLR) and platelet to lymphocyte ratio (PLR) than those without CA-AKI. It also exhibited more severe anemia including decreased hemoglobin and red blood cell count (all *p* < 0.05). The baseline RDW, albumin and PLR between the two groups were statistically significant different after PSM. Binary logistic regression analysis showed that baseline RDW, albumin and eGFR were correlated with CA-AKI after contrast-enhanced CT examination. The RDW exhibited moderated discrimination ability for predicting CA-AKI beyond eGFR, with an AUC of 0.803 (95% CI [0.702–0.90]) vs 0.765 (95% CI [0.70–0.83]).

**Conclusion:**

Increased baseline RDW and decreased eGFR are risk factors for CA-AKI after enhanced CT. RDW exhibited good predictive value and can be used as an early warning marker for patients suffering from CA-AKI after enhanced CT.

## Introduction

The application of contrast agents has remarkably increased the sensitivity of medical imaging, however, the intrinsic toxicity of contrast agents can also results in a series of adverse effects, including contrast associated acute kidney injury (CA-AKI). CA-AKI refers to a pathological condition that commonly occurs 48 h after iodine-based contrast exposure, an increase in the level of serum creatinine (SCr) of more than 44.2 umol/l or 25%, compared to the baseline SCr level (Kellum et al., 2012). With the broad application of iodine contrast agent in the field of radiation diagnosis and interventional therapy, the incidence of CA-AKI is still high and did not decrease with time (Lun et al., 2021). Currently, CA-AKI has become the third leading cause of acute renal failure in hospitals after insufficient renal blood perfusion and nephrotoxic drug treatment (Katzberg & Haller, 2006) and became one of the leading causes of iatrogenic renal insufficiency (Nash, Hafeez & Hou, 2002). Quoted rates of CA-AKI vary from 0% to 90% depending on the presence or absence of risk factors such as preexisting renal insufficiency, hypovolemia, the dose and type of radiographic contrast media used, diabetes mellitus, hypertension, advanced age, and concurrent intake of potentially nephrotoxic drugs (Song et al., 2014; Moos et al., 2013; Baker & Baker, 2001). In a meta-analysis of 15,582 patients who received contrast medium, the incidence of CA-AKI was 6.4% (McDonald et al., 2013).

Apart from periprocedural hydration, which seems to reduce but not completely prevent the risk of renal injury following radiographic contrast media (RCM) administration (Jurado-Roman et al., 2015; Solomon et al., 1994; Eisenberg, Bank & Hedgock, 1981), the results of other agents such as dopamine, mannitol, N-acetylcysteine, captoopril and statins are equivocal (Dabare et al., 2013; Weisbord & Palevsky, 2010; Verdoodt et al., 2018). Since there is no available treatment and even a mild 0.3 mg/dl increase of serum creatinine also associated with a 4.3-fold increase in the odds of death, it is urgent to find the valuable risk factors (Chertow et al., 2005). Risk assessment is traditionally based on the calculation of estimated glomerular filtration rate (eGFR), but it is not precise in the range beyond 60 ml/min. Hence, eGFR is unable to detect mild tubular injury.

The red blood cell distribution width (RDW) is calculated from the ratio of average red blood cell volume to its standard deviation, reflecting the variation degree of red blood cell volume. It is mainly used for differential diagnosis of different types of anemia (Lin et al., 1992). Recent studies have shown that increased RDW correlates with an increased death risk from sepsis, chronic heart failure, coronary heart disease, stroke and many other diseases (Kim et al., 2013; Felker et al., 2007; Tonelli et al., 2008; Ani & Ovbiagele, 2009). Some recent studies have indicated that RDW is an independent predictor (Zbierska-Rubinkiewicz et al., 2017; Mizuno et al., 2015) of acute kidney injury (AKI) after the percutaneous coronary intervention (PCI), but there is no report about the relationship between RDW and CA-AKI after enhanced CT. Hence, the aim of this study was to explore the novel risk factors for CA-AKI after enhanced computed tomography (CT) and analyze whether RDW can be used as an early predictive factor of CA-AKI after enhanced CT.

## Materials & Methods

### Research subjects

This is a retrospective study of hospitalized patients who underwent enhanced CT examination from 1 January 2017 to 1 December 2019 in Huizhou Central People’s Hospital. Inclusion criteria: (1) at least 18 years of age; (2) complete clinical data, including serum creatinine data one week before the enhanced CT examination and within three days after applying the contrast medium. Exclusion criteria: patients with severe heart failure (NYHA (New York Heart Association) heart function grade ≥3), acute coronary syndrome, dialysis, severe liver insufficiency, thyroid function disease, malignant tumor, autoimmune disease, blood system disease, severe infection and patients receiving antibiotics. The diagnostic criteria of CA-AKI were serum creatinine levels of ≥44.2 µmol/L or increasing to >25% of the baseline value (baseline value at admission) within three days after the application of contrast medium, and other renal function damage factors, such as intravascular volume depletion, hypotension, sepsis, and shock induced pre-renal kidney failure, post-renal kidney injury provoked by obstructive causes and use of nephrotoxic drugs (*i.e.*, non-steroidal anti-inflammatory drugs, penicillins, acetaminophen, diuretics, *etc.*) were excluded. Patients with CA-AKI after enhanced CT examination were assigned to the CA-AKI group, while those without CA-AKI were assigned to the non-CA-AKI group. This study was approved by the Medical Ethics Committee of Huizhou Central People’s Hospital (Approval Number:LLBA201951A).

### Clinical characteristics and laboratory data

Clinical characteristics at the time of admission were collected, including the age, sex, BMI, disease history (including kidney disease, hypertension, diabetes, stroke, cirrhosis, cardiac insufficiency and coronary heart disease), smoking status, systolic blood pressure, diastolic blood pressure, use of renin-angiotensin aldosterone system (RAAS) blockade, use of non-steroidal anti-inflammatory drugs, statins, and aminoglycoside antibiotics. The related data on the contrast medium were recorded, including the type, dose, route of administration, whether it was reused, the time interval of undergoing enhanced CT.

Laboratory data at the time of admission were also obtained from the medical records, including hemoglobin, white blood cell count, red blood cell count, red blood cell distribution width, platelet count, albumin, total cholesterol, triglyceride, high-density lipoprotein (HDL), low-density lipoprotein (LDL), uric acid, and serum creatinine. The serum creatinine value was rechecked three days after administering the contrast medium (if there were multiple check values, the highest value was taken). The estimated glomerular filtration rate (eGFR) was calculated by the CKD-EPI (Chronic Kidney Disease Epidemiology Collaboration) method.

### Statistical analysis

SPSS 24.0 was used for statistical analysis, and statistical significance was set at *P* < 0.05. The measurement data conforming to normal distribution were expressed by x ± s, and comparisons between the two groups were performed by *t*-test. The measurement data that did not exhibit normal distribution were expressed by M (1/4, 3/4), and Mann–Whitney U test was used for comparisons between the two groups. Count data were expressed by frequencies and percentages, and Fisher’s exact test was used for comparisons between the two groups. The propensity score method (PSM) was employed to control the deviation of baseline data between the two groups. The selected variables, including age, gender, weight, and smoking or not smoking, the four covariates were analyzed by PSM. The tendency scores of patients in each group were calculated by the 1:4 nearest neighbor matching method. To prevent too great matching differences of each group, caliper matching was adopted, and the logarithmic standard deviation of tendency score was limited to 0.02. Univariate and multivariate binary logistic regression analyses were utilized to evaluate the risk factors of CA-AKI after enhanced CT examination. The indexes with statistical differences in clinical data and laboratory data between the two groups, the basic diseases (hypertension, diabetes) with an impact on renal function and the dosage of contrast agent were included in the univariate regression analysis, the indexes with *P* < 0.05 were included in the multivariate conditional logistic regression analysis. The receiver operating characteristic (ROC) curve and the area under the curve (AUC) were used to evaluate the accuracy, sensitivity, and specificity of RDW in diagnosing CA-AKI after enhanced CT.

## Results

### Comparison of data between the two groups

A total of 632 cases were included, with 25 cases in the CA-AKI group and 608 cases in the non-CA-AKI group. The incidence of CA-AKI in the patients was 3.96% after enhanced CT examination. The age and proportion of smoking in the CA-AKI group were significantly higher than the non-CA-AKI group (*P* = 0.0002 and *P* = 0.027). There was no significant difference in male ratio, diabetes, hypertension, coronary heart disease, and contrast medium dosage between the two groups. Concerning laboratory data, baseline serum creatinine, RDW, NLR and PLR in the CA-AKI group were higher than those in the non-CA-AKI group (*P* < 0.0001), while eGFR (61.12 *vs* 84.38 ml/min, *P* < 0.0001), hemoglobin (112 *vs* 131 g/L, *P* = 0.0039), red blood cell count (4.12 ± 0.79 *vs* 4.42 ± 0.65, *P* = 0.021, and albumin (33.9 *vs* 39.5 g/L, P < 0.0001) at baseline were significantly lower than those in the non-CA-AKI group. No statistically significant differences were detected in total protein, total cholesterol, triglyceride, LDL, HDL, uric acid, and blood glucose between the two groups (Table 1). There were significant differences in baseline RDW, eGFR, serum albumin, and PLR between the two groups after PSM (P < 0.0001, *P* = 0.019, *P* = 0.005 and *P* = 0.009, Table 2).

**Table 1:** Comparison of the clinical and laboratory characteristics between patient groups. The table shows that the age and proportion of smoking in the CA-AKI group were significantly higher than the no-CA-AKI group. Concerning laboratory data, baseline serum creatinine, RDW, NLR and PLR in the CA-AKI group were higher than those in the no-CA-AKI group, while eGFR, hemoglobin, red blood cell count, white blood cell count, and albumin at baseline were significantly lower than those in the no-CA-AKI group.

**Variables**	**No-CA-AKI group (*n* = 607)**	**CA-AKI group (*n* = 25)**	***P* value**
Clinical characteristics			
Age [years]	61.00 (51.00, 71.00)	72 (61.00, 81.00)	0.0002
Male [n (%)]	375 (61.78)	17 (68.00)	0.675
Diabetes [n (%)]	123 (20.26)	4 (16.00)	0.800
Hypertension [n (%)]	268 (44.15)	11 (44.00)	1.000
Coronary heart disease [n (%)]	78 (12.85)	5 (20.00)	0.214
Smoking [n(%)]	138 (22.73)	11 (44.00)	0.027
Statins [n(%)]	225 (37.07)	7 (28.00)	0.405
ACEI/ARB [n(%)]	82 (13.51)	4 (16.00)	0.953
Total amount of contrast (ml, x ± s)	90.91 ± 16.12	85.93 ± 19.91	0.106
Laboratory characteristics			
Baseline serum creatinine (µmol/L, M(1/4, 3/4))	80.0 (65.00, 99.00)	95.0 (85.00, 130.50)	0.0002
Baseline eGFR (ml⋅ min^*symbol*−1^⋅ (1.73 m^**2**^)^*symbol*−1^)	84.38 (64.77,98.71)	61.12 (47.73,74.44)	<0.0001
Hemoglobin (g/L)	131.0 (119.0, 141.0 )	112.0 (93.0,138.5 )	0.0039
Red cell distribution width (%)	13.20 (12.70, 13.80)	15.10 (13.30, 16.35)	<0.0001
Red blood cell count(×10^**12**^/L)	4.42 ± 0.65	4.12 ± 0.79	0.021
White blood cell count (×10^**9**^/L)	7.90 (6.20, 10.20)	7.60 (5.35, 8.55)	0.115
Total serum protein (g/L, x ±s)	70.02 ± 6.89	68.46 ± 8.38	0.275
Albumin (g/L)	39.50 (36.00,42.90)	33.90 (30.80, 38.55 )	<0.0001
Total cholesterol (mmol/L)	4.58 (3.88, 5.37)	3.92 (3.43, 5.37)	0.150
Triglyceride (mmol/L)	1.23 (0.85, 1.83)	1.31 (0.73, 1.69)	0.699
LDL-C (mmol/L)	2.64 (2.04, 3.29 )	2.34 (1.93, 3.42 )	0.323
HDL-C (mmol/L)	1.00 (0.79, 1.24 )	0.94 (0.76, 1.06 )	0.233
Uric acid (µmol/L)	347.50 (268.00, 432.00)	357.00 (289.8, 465.00)	0.362
Glucose (mmol/L)	5.80 (5.00, 7.10)	5.40 (4.70, 6.60)	0.268
NLR	2.42 (1.77,3.51)	3.42 (1.87,5.81)	0.028
PLR	113.15 (84.24,150.83)	148.72 (111.51,304.22)	0.0006

**Notes.**

Abbreviations CA-AKI contrast-associated acute kidney injury eGFR estimated glomerular filtration rate ACEI/ARB angiotensin-converting enzyme inhibitor/angiotensin receptor blocker LDL-C high-density lipoprotein cholesterol HDL-C low-density lipoprotein cholesterol NLR neutrophil to lymphocyte ratio PLR platelet to lymphocyte ratio

**Table 2:** Comparison of the clinical and laboratory characteristics between patient groups after PSM. The table shows that there were significant differences in baseline RDW, serum albumin and PLR between the two groups after PSM.

**Variables**	**No-CA-AKI group (*n* = 100)**	**CA-AKI group (*n* = 25)**	***P value***
Clinical Characteristics			
Age (years)	71.5 (36.00, 89.00)	72 (40.00, 88.00)	0.793
Male [n (%)]	66 (66.00)	17 (68.00)	0.850
Diabetes [n (%)]	26 (26.00)	4 (16.00)	0.433
Hypertension [n (%)]	51 (51.00)	11 (44.00)	0.656
coronary heart disease [n (%)]	19 (19.00)	5 (20.00)	1.000
Smoking [n(%)]	43 (43.00)	11 (44.00)	1.000
Statins [n(%)]	45 (45.00)	7 (28.00)	0.173
ACEI/ARB [n(%)]	19 (19.00)	4 (16.00)	1.000
Total amount of contrast (ml, x ± s)	86.23 ± 17.48	85.93 ± 19.91	0.940
Laboratory characteristics			
Baseline serum creatinine (µmol/L, M(1/4, 3/4))	83.5 (66.0,113.3)	95.0 (85.00, 130.50)	0.020
Baseline eGFR (ml⋅ min ^*symbol*−1^⋅ (1.73 m ^**2**^) ^*symbol*−1^)	73.25 (52.65,87.52)	61.12 (47.73,74.44)	0.019
Hemoglobin (g/L)	126.0 (113.0, 137.0 )	112.0 (93.0,138.5 )	0.058
Red cell distribution width (%)	13.30 (12.70, 13.90)	15.10 (13.30, 16.35)	<0.0001
Red blood cell count (×10^**12**^/L)	4.24 ± 0.62	4.12 ± 0.79	0.411
White blood cell count (×10^**9**^/L)	8.10 (6.83, 10.30)	7.60 (5.35, 8.55)	0.053
Total serum protein (g/L, x ± s)	69.31 ± 8.12	68.46 ± 8.38	0.644
Albumin (g/L)	37.50 (34.90,40.53)	33.90 (30.80, 38.55 )	0.005
Total cholesterol (mmol/L)	4.34 (3.75, 5.26)	3.92 (3.43, 5.37)	0.527
Triglyceride (mmol/L)	1.21 (0.85, 1.64)	1.31 (0.73, 1.69)	0.888
LDL-C (mmol/L)	2.51 (1.91, 3.21 )	2.34 (1.93, 3.42 )	0.672
HDL-C (mmol/L)	0.96 (0.82, 1.13 )	0.94 (0.76, 1.06 )	0.573
Uric acid (µmol/L)	350.0 (283.0, 421.0)	357.00 (289.8, 465.00)	0.375
Glucose (mmol/L)	5.60 (5.13, 6.60)	5.40 (4.70, 6.60)	0.222
NLR	2.55 (1.86,3.88)	3.42 (1.87,5.81)	0.121
PLR	111.73 (85.82,180.46)	148.72 (111.51,304.22)	0.009

**Notes.**

Abbreviations CA-AKI contrast-associated acute kidney injury eGFR estimated glomerular filtration rate ACEI/ARB angiotensin-converting enzyme inhibitor/angiotensin receptor blocker LDL-C high-density lipoprotein cholesterol HDL-C low-density lipoprotein cholesterol NLR neutrophil to lymphocyte ratio PLR platelet to lymphocyte ratio

### Association between RDW and CA-AKI after enhanced CT

Univariate binary logistic regression analysis results showed that age (OR = 1.066, 95% CI [1.029–1.104], *P* < 0.001), smoking (OR = 2.067, 95% CI [1.185–6.016], *P* = 0.018), baseline serum creatinine (OR = 1.013, 95% CI [1.005–1.021], *P* = 0.002), baseline eGFR (OR = 0.964, 95% CI [0.948–0.981], *P* < 0.001), hemoglobin (OR = 0.957, 95% CI [0.936–0.978], *P* < 0.001), RDW (OR = 2.652, 95% CI [1.950–3.607], *P* < 0.001), red blood cell count (OR = 0.492, 95% CI [0.270–0.898], *P* = 0.021), serum albumin (OR = 0.831, 95% CI [0.771–0.896], P < 0.001), NLR (OR = 1.090, 95% CI [1.000–1.170], *P* = 0.024) and PLR (OR = 1.000, 95% CI [1.000–1.010], P < 0.001) were correlated with CA-AKI after enhanced CT examination. These ten variables were included in the multivariate analysis, and the results indicated that baseline eGFR (OR = 0.974, 95% CI [0.954–0.994], *P* = 0.014) and serum albumin (OR = 0.885, 95% CI [0.812–0.964], *P* = 0.005) levels were negatively correlated with CA-AKI after enhanced CT, while baseline RDW (OR = 2.246, 95% CI [1.616–3.122], *P* < 0.001) was positively correlated with it (Table 3). After PSM, the results of univariate binary logistic regression analysis indicated that serum albumin (OR = 0.874, 95% CI [0.797–0.951], *P* = 0.003), hemoglobin (OR = 0.974, 95% CI [0.951–0.995], *P* = 0.020), baseline RDW (OR = 3.135, 95% CI [1.994–5.456], *P* < 0.001), baseline eGFR (OR = 0.974, 95% CI [0.953–0.995], *P* = 0.018) and PLR ratio (OR = 1.000, 95% CI [1.000–1.010], P < 0.001) were correlated with CA-AKI after enhanced CT examination (Table 4). Thus, these variables were included in the multivariate analysis, and the results demonstrated that baseline RDW (OR = 2.929, 95% CI [1.512–5.674], *P* = 0.006) was positively correlated with CA-AKI after enhanced CT.

**Table 3:** Factors predicting contrast-induced nephropathy on logistic regression analysis. The table indicates that baseline eGFR and serum albumin levels were negatively correlated with CIN after enhanced CT, while RDW was positively correlated with it.

	Univariate analysis			Multivariate analysis		
Variables	*OR*	95% CI	*P* value	*OR*	95% CI	*P* value
Age (years)	1.066	1.029∼1.104	<0.001			
Diabetes (yes = 1, no = 0)	0.750	0.253∼2.2236	0.603			
hypertension (yes = 1, no = 0)	0.994	0.444∼2.225	0.988			
smoking (yes = 1, no = 0)	2.607	1.185∼6.016	0.018			
Baseline serum creatinine (µmol/L)	1.013	1.005∼1.021	0.002			
Baseline eGFR (ml⋅ min^−1^⋅ (1.73 m^2^)^−1^)	0.964	0.948∼0.981	<0.001	0.974	0.954∼0.994	0.014
Hemoglobin (g/L)	0.957	0.936∼0.978	<0.001			
Red blood cell distribution width	2.652	1.950∼3.607	<0.001	2.246	1.616∼3.122	<0.001
Red blood cell count	0.492	0.270∼0.898	0.021			
Albumin	0.831	0.771∼0.896	<0.001	0.885	0.812∼0.964	0.005
Uric acid	1.002	0.999∼1.005	0.180			
Total amount of contrast (ml)	0.976	0.931∼1.024	0.319			
NLR	1.090	1.000∼1.170	0.024			
PLR	1.000	1.000∼1.010	<0.001			

**Notes.**

Abbreviations eGFR estimated glomerular filtration rate NLR neutrophil to lymphocyte ratio PLR platelet to lymphocyte ratio

**Table 4:** Factors predicting contrast-induced nephropathy on Logistic Regression Analysis after PSM. The table demonstrates that after PSM, baseline RDW was positively correlated with CIN after enhanced CT.

	Univariate analysis			Multivariate analysis		
Variables	*OR*	95% CI	*P* value	*OR*	95% CI	*P* value
Age (years)	1.003	0.968∼1.044	0.863			
Diabetes (yes = 1, no = 0)	0.542	0.148∼0.158	0.300			
hypertension (yes = 1, no = 0)	0.755	0.307∼1.818	0.532			
smoking (yes = 1, no = 0)	1.042	0.423∼2.515	0.928			
Baseline serum creatinine (µmol/L)	1.011	0.999∼1.022	0.053			
Baseline eGFR (ml⋅ min^−1^⋅ (1.73 m^2^)^−1^)	0.974	0.953∼0.995	0.018	0.976	0.946∼1.006	0.117
Hemoglobin (g/L)	0.974	0.951∼0.995	0.020			
Red cell distribution width (%)	3.135	1.994∼5.456	<0.001	2.929	1.512∼5.674	0.006
Red blood cell count (%)	0.755	0.387∼1.480	0.408			
Albumin (g/L)	0.874	0.797∼0.951	0.003	0.928	0.817∼1.055	0.252
Uric acid (µmol/L)	1.003	0.999∼1.007	0.098			
Total amount of contrast (ml)	0.999	0.974∼1.024	0.939			
NLR	1.032	0.946∼1.112	0.428			
PLR	1.002	1.000∼1.004	0.074			

**Notes.**

Abbreviations eGFR estimated glomerular filtration rate NLR neutrophil to lymphocyte ratio PLR platelet to lymphocyte ratio

### The value of RDW in predicting CA-AKI after enhanced CT

**Figure 1:**
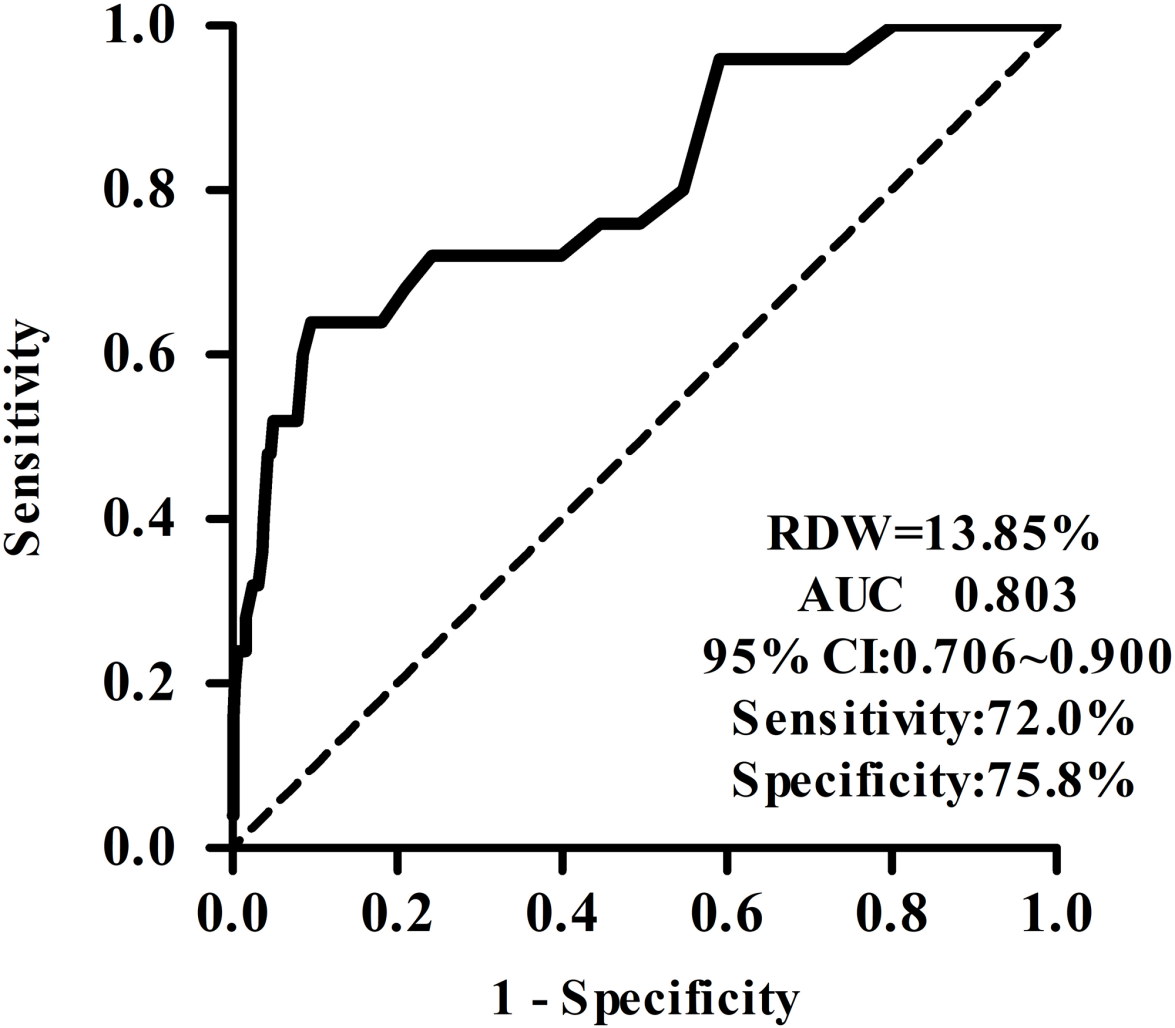
The value of RDW in predicting CIN after enhanced CT. ROC curve shows that the area under the curve for RDW predicting CIN after enhanced CT examination was 0.803, and with 13.85% as the cut-off point, the sensitivity and specificity of RDW in predicting CIN after enhanced CT examination were 72.00% and 75.80%, respectively.

ROC curve showed that the area under the curve (AUC) for RDW predicting CA-AKI after enhanced CT examination was 0.803, and with 13.85% as the cut-off point, the sensitivity and specificity of RDW in predicting CA-AKI after enhanced CT examination were 72.00% and 75.80%, respectively (Fig. 1). However, RDW improved the prediction of CA-AKI over eGFR with an AUC of 0.765 (95% CI [0.70–0.83] (Fig. S1).

## Discussion

Enhanced computed tomography is a scanning method by injecting an iodine contrast medium into the body quickly, which can show the scope and characteristics of lesions more clearly to improve the accuracy of imaging diagnosis. There are few studies on CA-AKI after enhanced CT examination. Fukushima et al. (2017) found that cardiac dysfunction and ICU admission may be risk factors for CA-AKI in patients with preexisting renal dysfunction. When the serum creatinine increased to ≥ 44 µmol/L or increased by >25% in two days, or the creatinine clearance decreased by >25% compared with that before using the contrast medium, it was considered CA-AKI, with an incidence of 22.1%. In this study, 632 patients undergoing enhanced CT examinations were included, 25 of which suffered from CA-AKI, with an incidence of 3.96%. It is acceptable based on the following reasons: (1) patients in various age groups were included. (2) nonionic low-osmolar contrast was used in all the patients undergoing enhanced CT, and most of them had a normal renal function. (3) The subjects were hospitalized patients, and it was relatively easy to evaluate renal function before the examination; therefore, CA-AKI was largely avoided.

CA-AKI is one of the leading causes of acute renal failure in hospitals. Although during the administration of isotonic contrast medium, the evaluation of renal function before enhanced CT and the implementation of preventive measures, such as adequate hydration, can reduce the incidence of CA-AKI after enhanced CT, it is still a significant complication after enhanced CT examinations. Hence, it is imperative to determine the predictive factors of CA-AKI. In this study, the median RDW value of the CA-AKI group was 15.10%, which was significantly higher than that of the non-CA-AKI group (13.40%). The reference range of RDW in this hospital is 0–15%. Multivariate analysis revealed that baseline RDW was positively correlated with the development of CA-AKI after enhanced CT examination, in line with findings of previous studies (Zbierska-Rubinkiewicz et al., 2017; Yamazoe et al., 2016). In this study, the ROC curve was employed to further evaluate the predictive ability of RDW for CA-AKI after enhanced CT. The results showed that AUC was 0.803. When 13.85% was considered the cut-off point, the sensitivity and specificity for CA-AKI prediction were 72.00% and 75.80%, respectively, indicating that RDW could better predict the development of CA-AKI after enhanced CT, consistent with the results reported by Akin et al. (2015).

The etiology of CA-AKI is complex. Some studies have reported that the cause is the direct toxic effect of the iodine contrast medium. The medium can aggravate the existing renal dysfunction; therefore, chronic renal insufficiency is an independent risk factor of CA-AKI, with eGFR <60 mL⋅ min-1⋅ (1.73 m^2^)^−1^ as the risk threshold for CA-AKI. Kim et al. (2010) followed up the patients with CA-AKI after CT examinations in CKD (chronic kidney disease) stages 3 to 5 and found that the proportion of renal replacement therapy in patients with CA-AKI increased in the subgroup with eGFR <30 mL⋅ min-1⋅ (1.73 m^2^)^−1^. Therefore, it was considered that eGFR <30 mL⋅ min-1⋅ (1.73 m^2^)^−1^ was a predictive factor of renal replacement therapy after CA-AKI. Multivariate logistic regression analysis showed that the baseline eGFR was negatively correlated with CA-AKI after enhanced CT examination, while the correlation between baseline serum creatinine and CA-AKI was not statistically significant. Currently, eGFR is the most commonly used indicator of renal function in clinical practice. Therefore, eGFR should be used to evaluate renal function before enhanced CT examinations. After PSM of baseline data, the results showed that baseline RDW was positively correlated with CA-AKI after enhanced CT.

Some other studies suggest that the pathogenesis of CA-AKI is related to inflammatory reactions, immune responses, and oxidative stress mediated by the iodine contrast medium. Inflammatory reactions and oxidative stress play an essential role in the development of CA-AKI (Mehran et al., 2004; Shaker, El-Shehaby & El-Khatib, 2010; Huang et al., 2021).

Systemic inflammation increases kidney vulnerability to the local inflammatory processes that are elicited by contrast medium reabsorption. Contrast medium causes the damage of renal endothelial and tubular epithelial cells, triggers the generation of various cytokines and chemokines, then induces the recruiting of inflammatory cells into the kidney (Akcay, Nguyen & Edelstein, 2009). In recent years, some studies have shown that RDW is correlated with inflammatory reaction indexes (C-reactive protein and erythrocyte sedimentation rate) (Lippi et al., 2009), and pro-inflammatory factors, such as tumor necrosis factor- *α* (TNF-α) and interleukin-6 are positively correlated with RDW (Semba et al., 2010). The mechanism for an increase in RDW caused by inflammatory reactions might be related to the functions of inflammatory factors in reducing the sensitivity of bone marrow erythroid stem cells to erythropoietin stimulation, inhibiting their anti-apoptosis and promoting maturation (Patel et al., 2009). Inflammatory cytokines inhibit the maturation of red blood cells, resulting in the migration of immature red blood cells into the circulation, different sizes of red blood cells, and increased RDW. This study is retrospective and does not include inflammatory reaction indexes such as high-sensitivity C-reactive protein, erythrocyte sedimentation rate, and interleukin-6. To further explore the mechanism for the development of CA-AKI after enhanced CT, it is necessary to expand the sample size and detect the corresponding inflammatory factors. Second, some studies have shown that CA-AKI is due to oxidative stresses, which can increase the fragility of red blood cells (Tozzi-Ciancarelli et al., 1989), reduce their maturation rate (Marinkovic et al., 2007), shorten their survival time, and increased RDW. Therefore, an increase in RDW might indicate oxidative stress. Furthermore, the RDW value can reflect the nutritional status of patients. The results of this study showed that the RDW value of the CA-AKI group was higher, and its albumin level was lower. The binary logistic regression analysis showed that albumin level was negatively correlated with the development of CA-AKI after enhanced CT examinations because severe hypoproteinemia can lead to insufficient effective renal perfusion pressure and further result in CA-AKI.

There are some limitations in our study. First, the study was a single-center retrospective study with a small sample size, so multi-center investigations with larger sample sizes of enhanced CT cases need to be performed in the future. Second, further studies are needed to explore the association between RDW and hypoproteinemia. Third, relevant inflammatory factors should also be detected to further explore the mechanism for CA-AKI after enhanced CT examinations.

## Conclusion

In conclusion, we found that higher RDW could be independently associated with the risk of CA-AKI in patients undergoing enhanced CT examinations. This finding suggests that RDW presents a good predictive value and can be used as an early warning marker for CA-AKI after enhanced CT examination.

##  Supplemental Information

10.7717/peerj.14224/supp-1 Supplemental Information 1 Raw data

10.7717/peerj.14224/supp-2 Supplemental Information 2 The value of baseline eGFR in predicting CIN after enhanced CT ROC curve showed that the area under the curve for baseline eGFR predicting CIN after enhanced CT examination was 0.765.

10.7717/peerj.14224/supp-3 Supplemental Information 3 1:4 psm data

## Additional Information and Declarations

### Data Availability

The following information was supplied regarding data availability: The raw measurements are available in the Supplementary Files.
