# Supplementary figures and images for: A novel risk factor of contrast associated acute kidney injury in patients after enhanced computed tomography: a retrospective study
Source: PeerJ. 2022 Oct 20;10:e14224. doi: 10.7717/peerj.14224 (PMC9588300; doi:10.7717/peerj.14224)

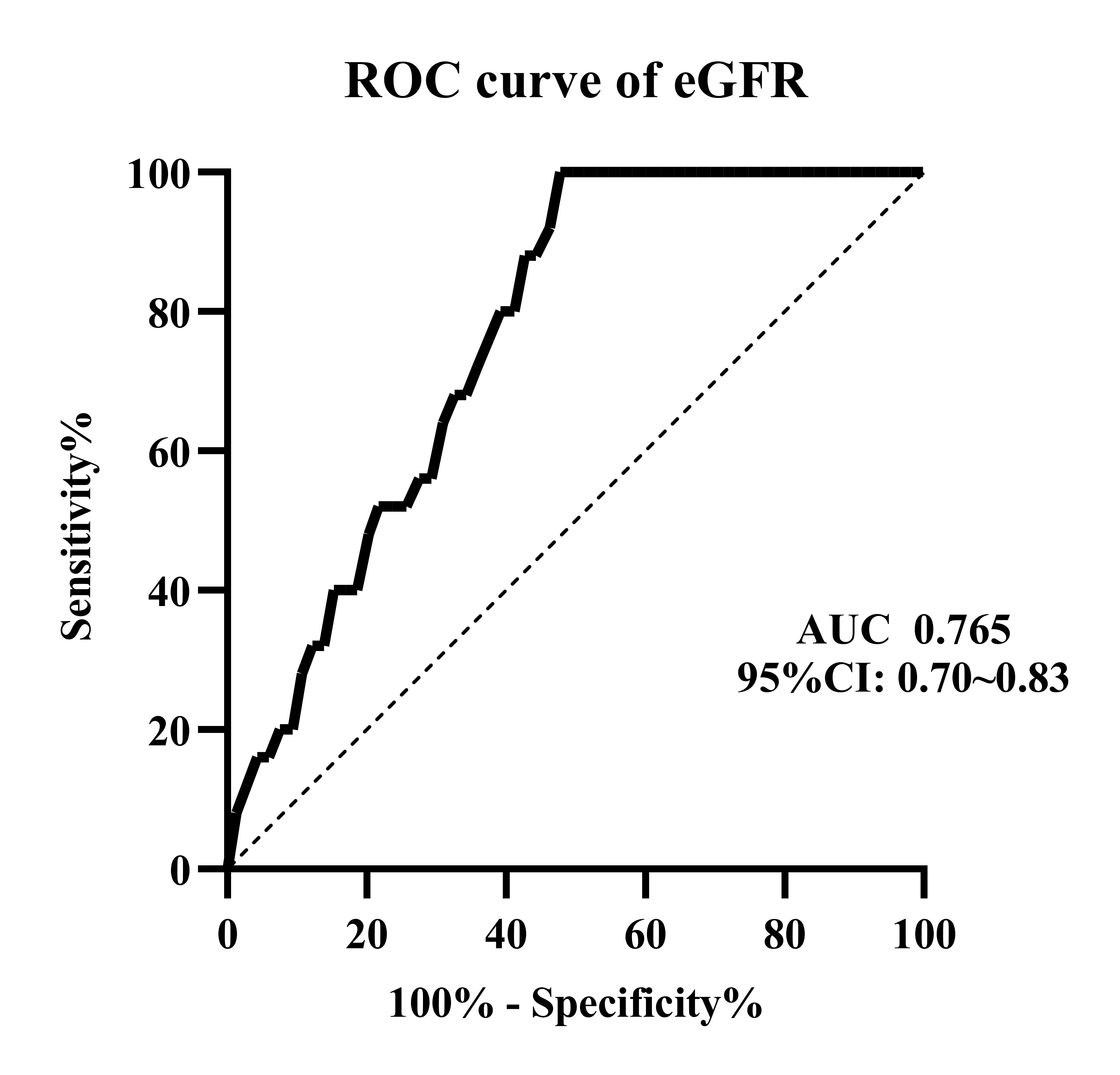

Supplement: Supplemental Information 2 — ROC curve showed that the area under the curve for baseline eGFR predicting CIN after enhanced CT examination was 0.765. [file peerj-10-14224-s002.tif]
